# The development of an open-access database for human transcriptional regulation interactions

**DOI:** 10.1186/1471-2105-12-S11-A10

**Published:** 2011-11-21

**Authors:** Luiz Augusto Bovolenta, Marcio Luis Acencio, Ney Lemke

**Affiliations:** 1 Department of Physics and Biophysics, Instituto de Biociências de Botucatu, Unesp -Univ Estadual Paulista, Botucatu -SP, Brazil

## Background

The modeling of interactions among transcription factors (TFs) and their respective target genes (TGs) into transcriptional regulatory networks is important for the complete understanding of regulation of biological processes. In the case of human TF-TG interactions, there is no database at present that explicitly provides such information even though many databases containing human TF-TG interaction data have been available, such as TRANSFAC [1] and TRED [2]. In an effort to provide researchers with a repository of TF-TG interactions from which such interactions can be directly extracted, we present here the human transcriptional regulation interactions database (HTRIdb), an open-access database of experimentally validated interactions among human TFs and their respective TGs.

## Materials and methods

The HTRIdb is implemented as a relational database PostgreSQL that is connected to a web interface via the JBOSS AS application server that dynamically generates user-friendly HTML front-end queries using the Apache Tomcat web server. For the visualization of TF-TG interactions, we embedded in the HTRIdb the Cytoscape Web.

## Results

The HTRIdb offers several mechanisms of data query and extraction, such as download in spreadsheet or text format and the visualization of TF-TG interactions. There is an update mechanism that allows scientists to send new data. The HTRIdb currently holds a collection of 2,114 unique transcriptional regulation interactions among 163 TFs and 1,034 TGs.

## Conclusion

HTRIdb is a powerful user-friendly tool from which human TF-TG interactions can be easily extracted.
